# Reproducibility of the computational fluid dynamic analysis of a cerebral aneurysm monitored over a decade

**DOI:** 10.1038/s41598-022-27354-w

**Published:** 2023-01-05

**Authors:** Phani Kumari Paritala, Haveena Anbananthan, Jacob Hautaniemi, Macauley Smith, Antony George, Mark Allenby, Jessica Benitez Mendieta, Jiaqiu Wang, Liam Maclachlan, EeShern Liang, Marita Prior, Prasad K. D. V. Yarlagadda, Craig Winter, Zhiyong Li

**Affiliations:** 1 School of Mechanical, Medical and Process Engineering, Faculty of Engineering, Queensland University of Technology, Brisbane, 4000 Queensland Australia; 2 Centre for Biomedical Technologies, Queensland University of Technology, Brisbane, Queensland Australia; 3 School of Mathematical Sciences, Queensland University of Technology, Brisbane, Queensland Australia; 4 The Kenneth G Jamieson Department of Neurosurgery, Royal Brisbane and Women’s Hospital, Brisbane, Queensland Australia; 5 Department of Medical Imaging, Royal Brisbane and Women’s Hospital, Brisbane, Queensland Australia; 6 Faculty of Sports Science, Ningbo University, Ningbo, China; 7 School of Chemical Engineering, University of Queensland, Brisbane, Queensland Australia

**Keywords:** Biomedical engineering, Aneurysm

## Abstract

Computational fluid dynamics (CFD) simulations are increasingly utilised to evaluate intracranial aneurysm (IA) haemodynamics to aid in the prediction of morphological changes and rupture risk. However, these models vary and differences in published results warrant the investigation of IA-CFD reproducibility. This study aims to explore sources of intra-team variability and determine its impact on the aneurysm morphology and CFD parameters. A team of four operators were given six sets of magnetic resonance angiography data spanning a decade from one patient with a middle cerebral aneurysm. All operators were given the same protocol and software for model reconstruction and numerical analysis. The morphology and haemodynamics of the operator models were then compared. The segmentation, smoothing factor, inlet and outflow branch lengths were found to cause intra-team variability. There was 80% reproducibility in the time-averaged wall shear stress distribution among operators with the major difference attributed to the level of smoothing. Based on these findings, it was concluded that the clinical applicability of CFD simulations may be feasible if a standardised segmentation protocol is developed. Moreover, when analysing the aneurysm shape change over a decade, it was noted that the co-existence of positive and negative values of the wall shear stress divergence (WSSD) contributed to the growth of a daughter sac.

## Introduction

Intracranial aneurysm (IA) is a cerebrovascular disorder that affects 2–5% of the world’s population^1–3^. It is an abnormal dilation of the arterial wall that often forms at bifurcation points of cerebral arteries^4^. Most aneurysms do not rupture and are typically small and asymptomatic^5^. However, aneurysm rupture is detrimental as it can lead to subarachnoid haemorrhage (SAH) which has a high rate of patient morbidity and mortality^6^.

There is still a limited understanding of the mechanisms of aneurysm formation, growth and rupture. According to current literature, intracranial aneurysm initiation is a gradual process influenced by genetic, environmental and hemodynamic risk factors^7^. Anatomical variations initiated by vessel wall degeneration alter the local hemodynamic flow contributing to structural remodelling and aneurysm growth^8,9^.

Unruptured intracranial aneurysms (UIAs) develop over the patient’s lifetime and are often discovered incidentally. UIAs can remain unchanged for a long period or undergo rapid growth with an increased risk of rupture^10,11^. The management of UIAs is a challenging clinical decision that is made by weighing the risk of rupture and the risk of intervention^12,13^. To stratify these risks, several guidelines have been developed using large-scale clinical studies. These include the PHASES (population, hypertension, age, size, earlier subarachnoid haemorrhage) score^14^, the UIATS (unruptured intracranial aneurysm treatment site) score^15^ and the ELAPSS (earlier subarachnoid haemorrhage, location of aneurysm, age, population, size and shape) score^16^. The accuracy of these scores was found to be suboptimal as quantitative factors such as blood flow haemodynamics and vascular wall biomechanics were not considered.

It is widely discussed in the literature that local flow dynamics contribute to the initiation, growth and rupture of cerebral aneurysms^17,18^. Hence, image-based patient-specific computational fluid dynamics (CFD) simulations are frequently used to understand the relationship between flow parameters and anatomical variations^18–22^. However, the simulated results are highly variable and are influenced by the imaging modality used^23,24^, segmentation and reconstruction technique^25,26^, rheology of blood flow, mesh size, time step, boundary conditions applied and operator-to-operator variability^27,28^.

These variations were demonstrated by a study by Geers et al. which compared CFD simulations of ten aneurysms imaged with 3D rotational angiography (3DRA) and computed tomography angiography (CTA)^23^. A large difference was found in the flow rates at the aneurysm neck (33.9 ± 7.6%) and the wall shear stress (WSS) (31.4 ± 9.9%)^23^. A later study by Ren et al. compared ten cerebral aneurysms imaged with MRA, CTA and 3DRA to assess the reproducibility of CFD simulations^24^. No significant differences were found when comparing the morphologies of CTA and 3DRA (8.3 ± 1.72%) and MRA and 3DRA (6.6 ± 1.85%). However, there was a large variation in the average WSS values when comparing CTA and 3DRA (34 ± 5.13%) and MRA and 3DRA (40.6 ± 9.21%). Small geometric variations significantly altered the flow field and key hemodynamic parameters such as vorticity, positive circulation, and wall shear stress^25^.

The Multiple Aneurysms Anatomy Challenge 2018 (MATCH) was conducted to better understand the impact of intracranial aneurysm segmentation on the simulated vessel haemodynamics. This challenge involved 26 research groups that used state-of-the-art segmentation on five IAs in a single patient scan. The first study compared the segmentation approaches used and observed inter-group differences of up to 20% in the aneurysm volume and 30% in the surface area^26^. The second study compared the effect of morphological parameters on hemodynamics and observed a difference of 30% and 46% in the mean aneurysmal velocity and the neck inflow rate respectively. A variation of 28–51% was found when comparing the time-averaged WSS (TaWSS) of the aneurysm^28^. The 2015 International Aneurysm CFD challenge quantified the real-world variability of the segmentation, reconstruction and CFD simulations between 26 research teams and observed a wide variability in techniques, model extents, inflow rates and blood properties used by different teams. Segmentation, reconstruction and boundary conditions influenced the hemodynamic parameters by up to 56%^27^.

As discussed, various reproducibility studies have been conducted between teams, however, there is a need to better understand the variability within a team given the same protocol^29^. New approaches for reducing the variability in the hemodynamic parameters should be considered to enable the translation of CFD results into clinical practice. The purpose of this study is to evaluate the intra-team reproducibility of model generation and its influence on the hemodynamic parameters for an intracranial aneurysm of one patient imaged six times over a decade. This study hypothesises that the variability in segmentation and reconstruction within a team given the same protocol contributes to the differences in the output hemodynamic parameters.

## Materials and methods

This study was approved by the institutional research ethics committee of Queensland University of Technology (1900000505, Project ID:2490) and the RBWH Ethics Board (LNR/2019/QRBW/49363). A waiver of consent for the de-identified retrospective patient-specific imaging data was obtained from the RBWH Ethics Board and the department of public health. All procedures performed were in accordance with the ethical standards of the Declaration of Helsinki.

### Patients and imaging

A patient with an unruptured middle cerebral artery (MCA) IA imaged at the Royal Brisbane and Women’s Hospital (RBWH) was the subject of investigation in this study. Six time-of-flight magnetic resonance angiography (TOF-MRA) scans of the brain were acquired at regular intervals over a decade to track its development (I-2010, II-2013, III-2014, IV-2015, V-2017, IV-2019). The resolution of these scans ranged from 2 to 3.4 pixels per mm.

### 3D geometry reconstruction

Cranial Time-of-flight (TOF) MRAs of the aneurysms were imported into the image processing software package, Amira (version 6.0, FEI, at Hillsboro, Oregon, USA). The patient images were segmented using a thresholding method to 3D reconstruct the MCA aneurysm. The region of interest for these models includes the aneurysmatic region and attached vessels. The geometries created from Amira were manually smoothed using Meshmixer 3.5 (2020 Autodesk, Inc) to improve the quality of the reconstruction by increasing the element density for the meshing process and improving CFD outcomes.

### Hemodynamic simulations

CFD simulations were performed using ANSYS Workbench (version 2020 R1, ANSYS, Canonsburg, PA, USA). The 3D aneurysm model was pre-processed using SpaceClaim and meshed with tetrahedral elements. The inbuilt ANSYS adaptive mesh was used to mesh all geometries and based on the mesh independence study a mesh size of 0.15 mm. A non-slip boundary condition of 10-layer inflation with a growth factor of 1.2 was used for all CFD simulations. Blood was set as a Newtonian fluid with properties of density as 1050 kg/m^3^ and viscosity of 0.00345 Pa s^30^. Blood flow was assumed as laminar incompressible and is governed by the Navier–Stokes equation. The time step for the simulations was 0.0005 s running for one cardiac cycle for all timepoints. The inlet and outlets were extended by 15 mm for a developed flow at the region of interest. A time-dependent pulsatile mass flow rate profile was defined at the inlet and constant pressure of 0 Pa^31^ was defined at the outlets. The inlet mass flow rate profile is included in the supplementary material (see Fig. S1 online).

### Reproducibility and follow-up study

Four operators of different skill levels (A, B, C, and D) were involved in this study. All operators are biomedical engineers with a baseline knowledge of human anatomy. Operator D is the experienced operator that developed the study protocol and trained all other operators. This training included a review into current literature to better understand the theory behind segmenting and modelling human vasculature.

Once the operators were familiar with the process, they were instructed to follow the same protocol for segmentation, reconstruction and CFD modelling. All operators were provided with six longitudinal datasets of TOF MRA scans of the MCA aneurysm. During the process the operators worked independently and were not given additional guidance by the trainer (Operator D).

Parameters for thresholding, the extent of smoothing and the length of the parent vessels were left to the operators’ discretion. Geometric and hemodynamic variability was measured to understand the effect of individuals' performance on the reproducibility of the hemodynamic quantities. In addition to the reproducibility, the models generated by operator D at the different time points were compared to analyse the shape changes in the investigated aneurysm and their influence on the CFD parameters.

### Data analysis

#### Geometric comparison

The geometries were compared using the deviation tool in Ansys 2020 R1 Spaceclaim and Geomagic Wrap 2021 (3D Systems, Inc.). To investigate the reproducibility of the geometry reconstruction the subsequent comparisons have been done (A–B; A–C; A–D; B–C; B–D; C–D). The first operator mentioned is the source and the second is the target (Source to Target). Switching the comparison order would only produce a negative difference and so was not investigated. Colour contours are plotted with a tolerance of 0.01 mm represented in green, inside tolerance (IT) represented in blue and outside tolerance (OT) represented in red. Root mean square (RMS) estimates of the deviations in geometry were extracted from Geomagic Wrap [2021 3D Systems, Inc.]. To investigate the shape changes at different timepoints the following comparisons were done for geometries reconstructed by operator D (Timepoint I–II; II–III; III–IV; IV–V; V–VI).

#### Hemodynamic comparison

CFD post (version 2020 R1, ANSYS, Canonsburg, PA, USA) and Tecplot 360 (2020 R2) were used to extract velocity streamlines, wall shear stress (WSS), time-averaged wall shear stress (TaWSS), oscillatory shear index (OSI), relative residence time (RRT) and wall shear stress divergence (WSSD) contours. The variation in the WSS, TaWSS, OSI and WSSD were analysed to characterise the relationship between changes in geometry and WSS. RStudio (1.4.1106; R 4.0.4)^32^ is used to plot the probability distributions of the WSS and WSS-derived parameters. The distribution of WSS per quartile and hemodynamic dependency per timepoint were calculated.

## Results

### Geometric reproducibility

**Figure 1:**
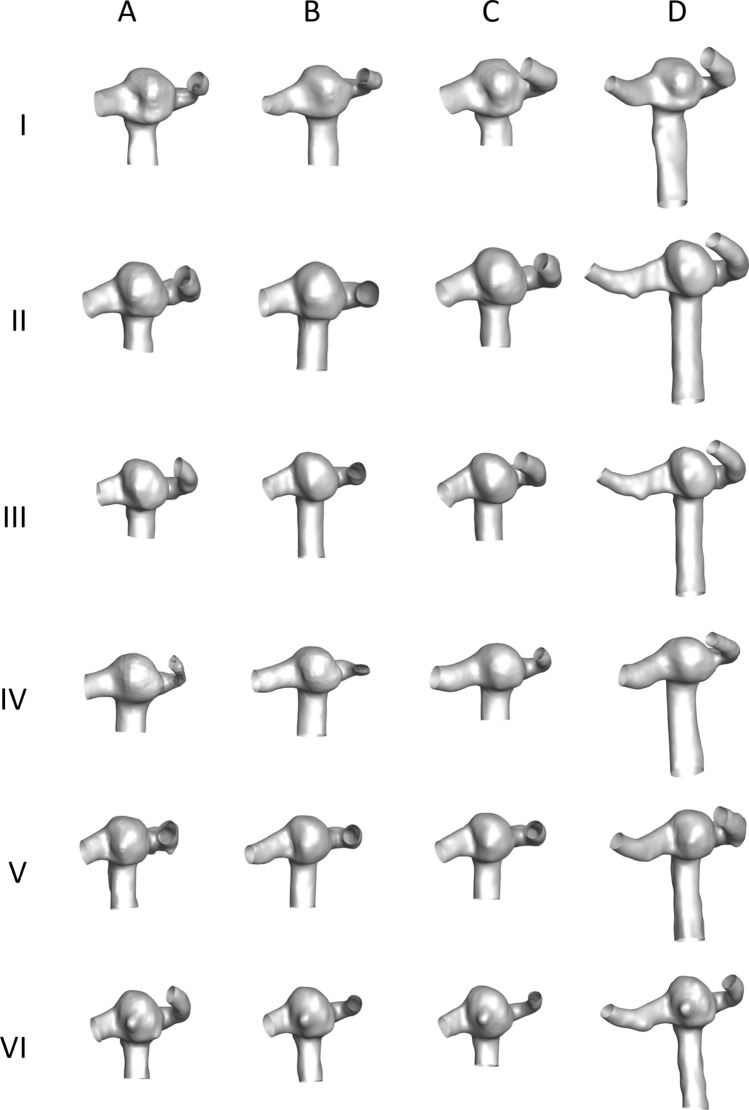
Reconstructed aneurysm geometries. I–VI indicate the time points and A–D indicates operators.

The variability in geometries reconstructed by different operators is shown in Fig. 1. From visual observation, the geometries reconstructed by operator A were found to be smoothed more than the geometries reconstructed by operators B, C and D. This can be observed in geometries modelled by operator A at timepoints I and IV where the outflow branches are narrower compared to operators B, C and D. The inlet and outflow branches were found be longer for operator D’s geometries compared to the other operators. However, as the geometries were extended for fully developed flow, this should not impact the results significantly.

**Figure 2:**
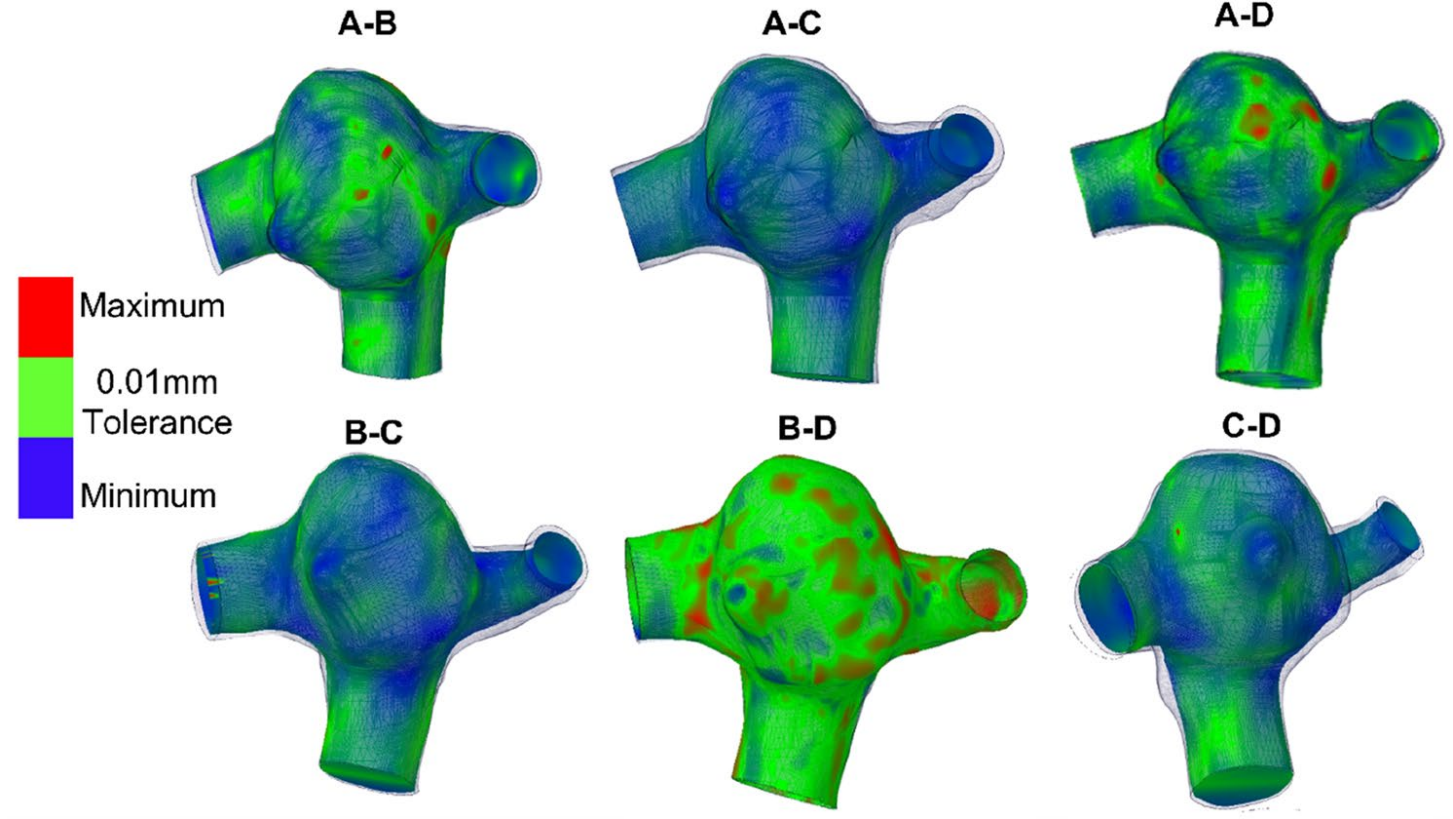
Contours representing the differences in surface roughness of the geometries for timepoint VI between operators. Green represents a tolerance of 0.01 mm, blue and red represent the minimum (inner tolerance, IT) and maximum variation (outer tolerance, OT) of the source geometry with respect to target geometry.

The deviation analysis tool in Ansys Spaceclaim has been used to identify the differences in surface roughness of the geometries reconstructed by different operators. Figure 2 represents the deviation of the region of interest between operators for one timepoint (VI). As shown the maximum inner tolerance of 0.51 mm is between operators A and C. and the maximum outer tolerance of 0.29 mm is between operators B and C.

### Hemodynamic reproducibility

**Figure 3:**
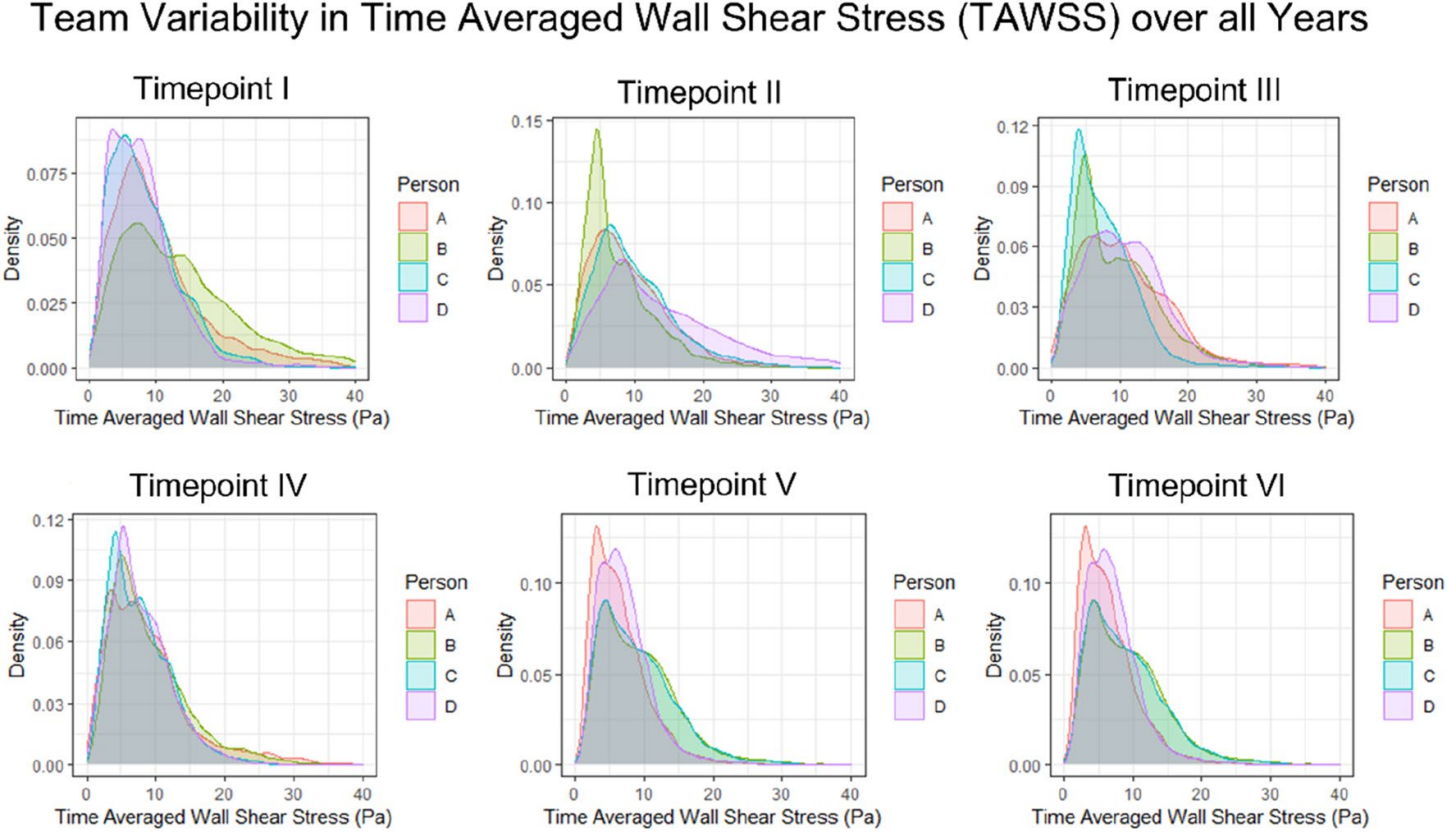
Density distribution plots representing the operator variability in time-averaged wall shear stress distribution at different time points.

The simulated results were analysed to understand the reproducibility of CFD parameters. Time-averaged wall shear stress (TaWSS) is the quantity of interest as it is most widely used in the literature for comparison. Additional hemodynamic reproducibility comparisons are included in the supplementary material (see Figs. S2–S7 online). The density distribution of TaWSS over one cardiac cycle between operators at different timepoints is shown in Fig. 3. The average overlap of the density distributions of TaWSS between different operators for all timepoints is A–B: 0.79, A–C: 0.89, A–D: 0.85, B–C: 0.70, B–D: 0.66 and C–D: 0.94. Overall TaWSS density distribution comparisons have resulted in 80% reproducibility between different operators with major variations corresponding to the differences in lengths of the inlet and outflow branches, the extent of smoothing and sharp corners due to inconsistent smoothing.

**Figure 4:**
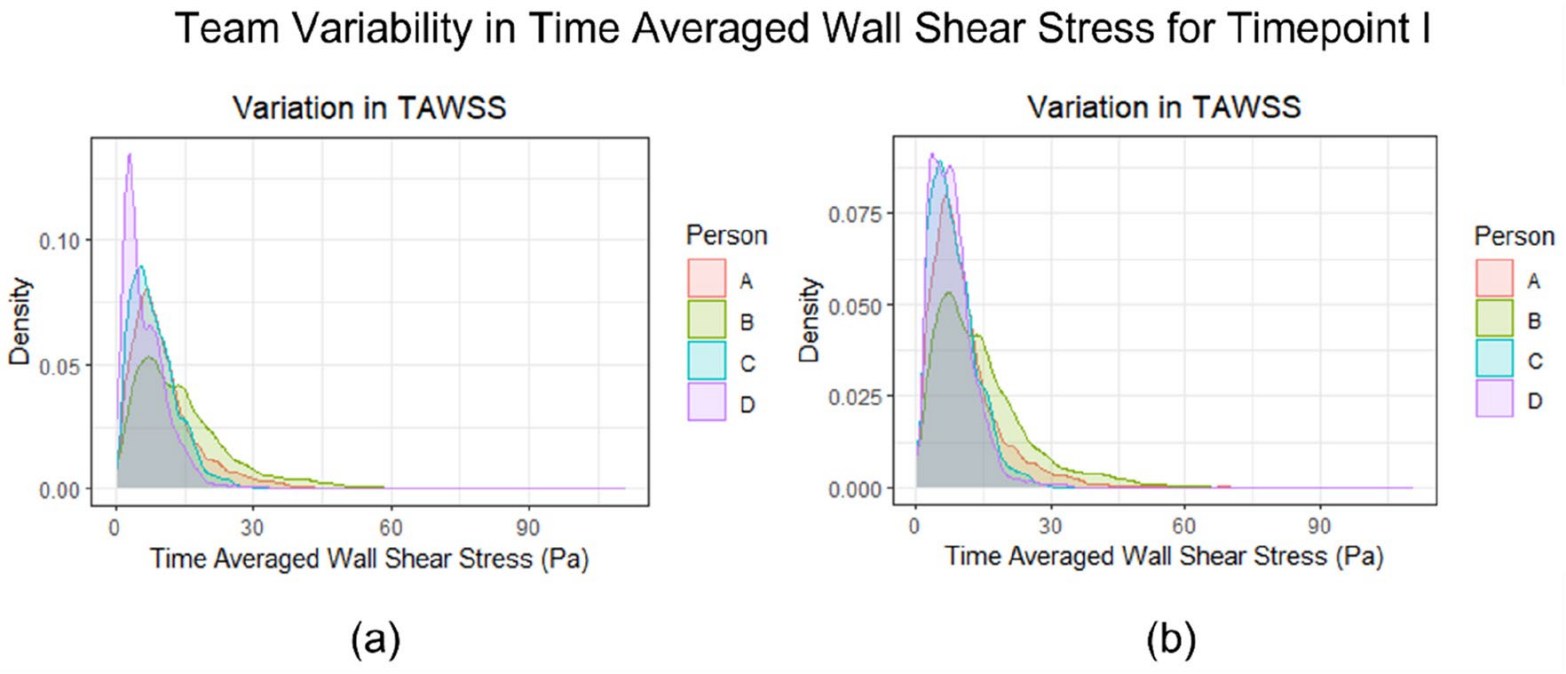
Comparison of the density distribution plots representing the operator variability in the TaWSS parameters for time point I. (**a**) Geometries with differences in the length of inlet and outflow branches as reconstructed by different operators; (**b**) post-processed geometries considering the region of interest (aneurysmatic region and attached arteries) for all operators.

To better understand the impact of the differences in the inlet and outlet lengths on the TaWSS, the density distributions were plotted for the entire geometry (Fig. 4a) and the region of interest (Fig. 4b) for all operators. When considering the entire geometry (Fig. 4a), the longer lengths of inlets and outlets of operator D result in the higher density distribution of TaWSS particularly in the lower range of values. Conversely, for the region of interest, the lower TaWSS density distribution of operator A is attributed to the shorter inlets and outlets and over-smoothing of the geometries.

**Figure 5:**
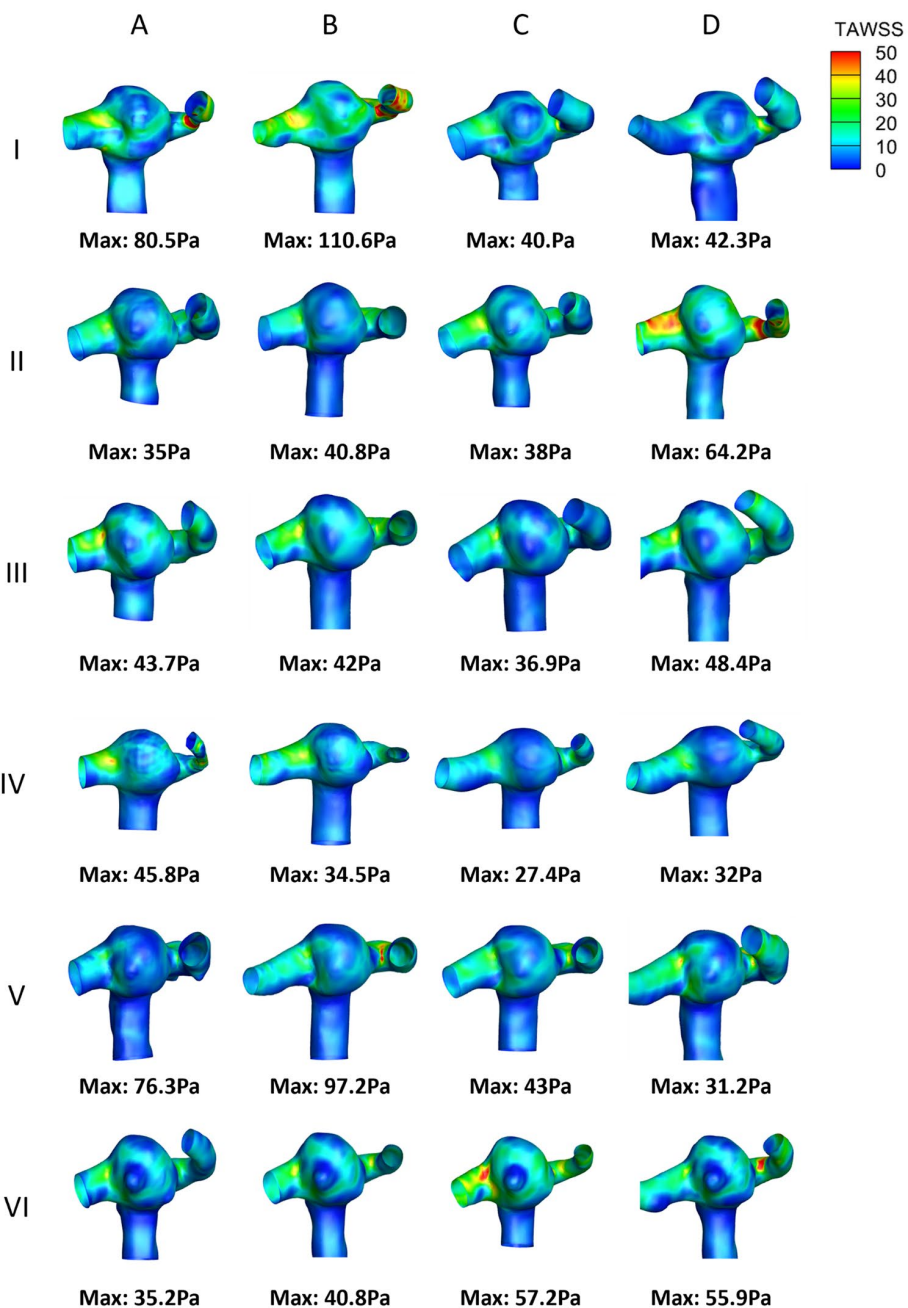
Representative colour contours (region of interest) showing the normalised Time-averaged wall shear stress for different operators at different time points. The maximum value of the TaWSS (red colour on the contour map) for each model is presented below in the image.

The normalised TaWSS distribution for different operators at different time points is shown in Fig. 5. The stress distributions were influenced by the extent of smoothing on the geometries. The level of smoothing also varied the location and magnitude of the maximum TaWSS. At timepoint I, sharp edges in operator B’s geometry result in a higher maximum TaWSS compared to other operators. This is likely due to poor quality mesh that led to a stress concentration at that location. Likewise, the maximum TaWSS is higher for A in contrast to C and D due to the over-smoothing of the geometry at the outflow branch. WSS and TaWSS density distribution comparisons have resulted in 80% reproducibility between different operators.

**Figure 6:**
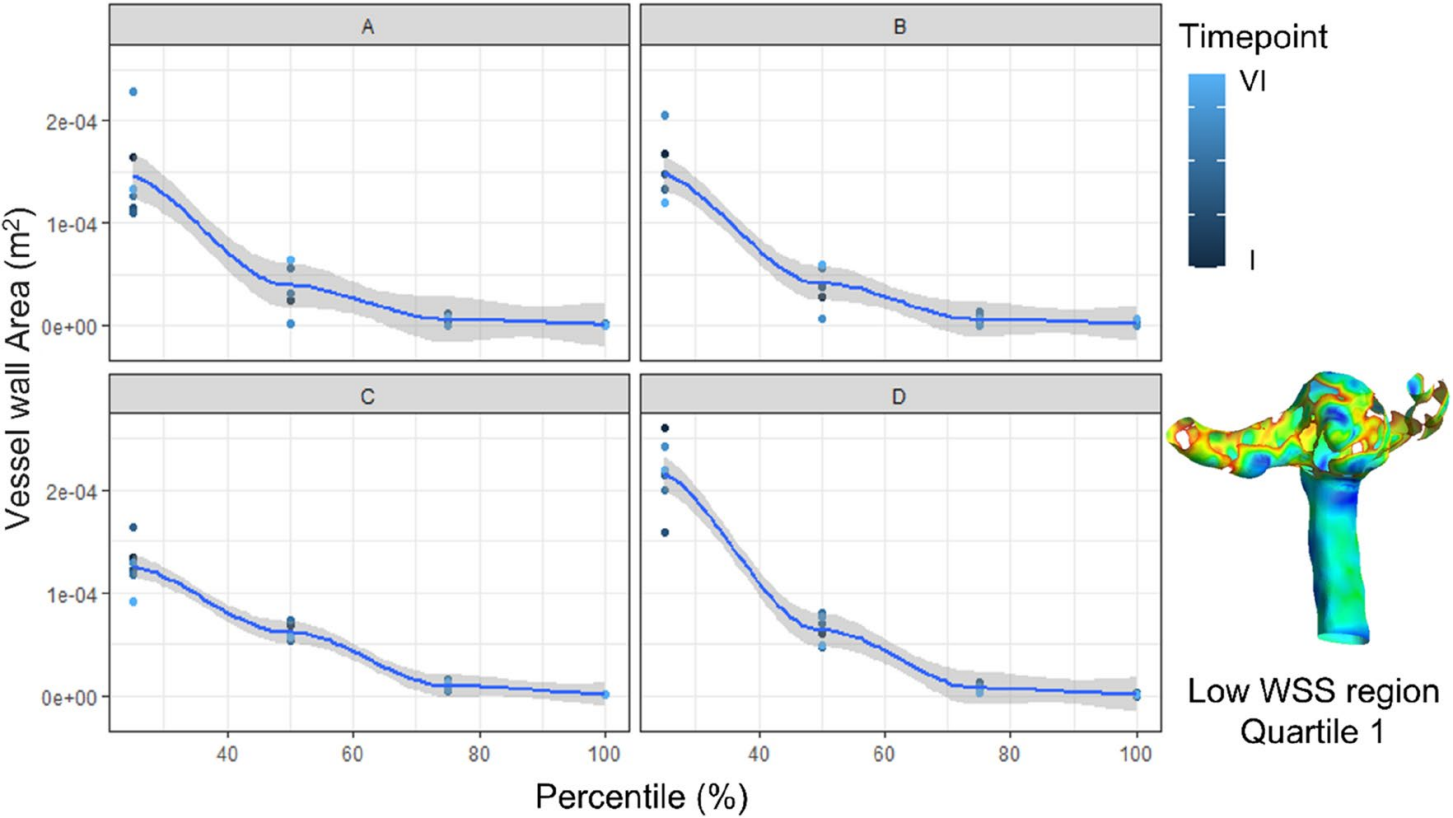
Difference in WSS area for geometries reconstructed by different operators at different timepoints. The contour represents an example of the first quartile of WSS.

In addition, Fig. 6 shows the differences in the WSS area for each quartile of WSS values for all operators at different time points. The differences in the vessel wall area corresponding to the first and second quartiles of the WSS distribution are higher compared to the third and fourth quartiles for each operator. This deviation is attributed to the differences in the length of inlet and outflow branches reconstructed by the operators.

### Geometric and hemodynamic variability between follow-up scans

**Figure 7:**
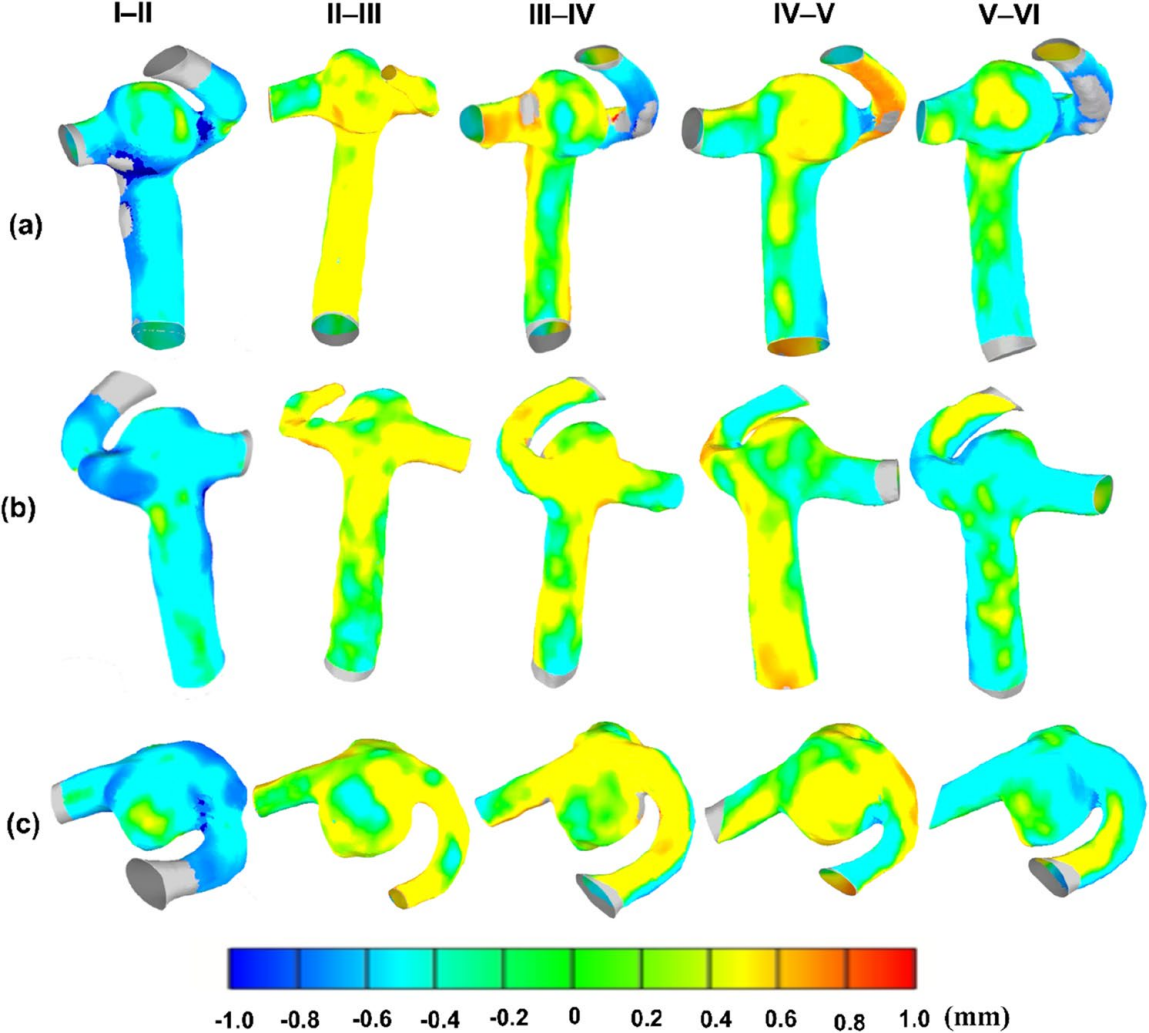
Colour contours representing the differences in the shape and size of the aneurysm over a decade. (**a**) Front view; (**b**) Back view; (**c**) Top view; I–II: Timepoint II compared to timepoint I with timepoint I as reference.

Figure 7 illustrates the changes in aneurysm shape and size at different timepoints. Given the small variability between operators, the geometries of operator D were used for the follow-up analysis as they developed the protocol and trained all the operators. The yellow contour highlights the localized area of the aneurysm where there are positive size changes, and the blue region shows the negative size change. Shape changes have been observed in regions relating to the changes in the blood flow direction, velocity and stress values.

**Figure 8:**
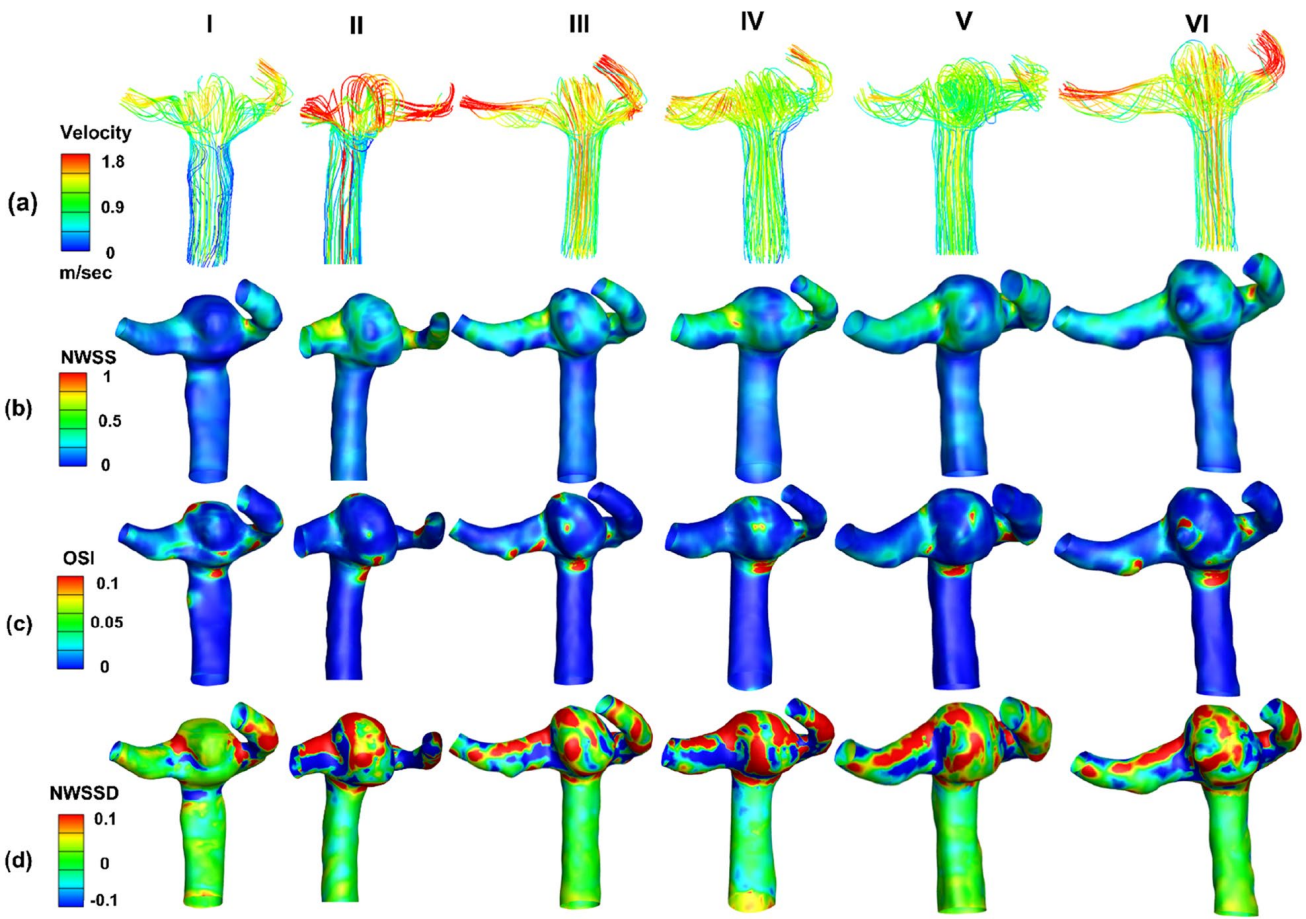
Contour plots for different timepoints at systole: (**a**) Velocity streamlines; (**b**) Normalised wall shear stress; (**c**) Oscillatory shear index; (**d**) Normalised wall shear stress divergence.

Figure 8a represents the velocity streamlines for the aneurysms at different time points at systole. It was observed that the blood flow in the aneurysm head is recirculating before going to the outflow branches. Also, the location of the maximum velocity is at the outflow branches where a reduction in vessel diameter has been observed. Figure 8b represents the normalised WSS distribution with maximum value in the outflow branches. Figure 8c represents the OSI distribution with the highest values within the dome and around the daughter sac. Figure 8d illustrates the distribution of normalised WSSD. The red regions represent the positive value of WSSD^+^ where the aneurysm wall is stretched. Blue regions represent the negative value of WSSD^−^ which shows compression on the aneurysm wall. WSSD^+^ and WSSD^−^ co-exist on the aneurysm surface due to recirculating blood flow. The distribution of WSSD in the earlier timepoints promotes aneurysm growth. The change in direction of WSS on the aneurysm surface at timepoint V caused the formation of a daughter sac that is visible in timepoint VI. This may eventually lead to the rupture of the aneurysm wall^33,34^.

**Figure 9:**
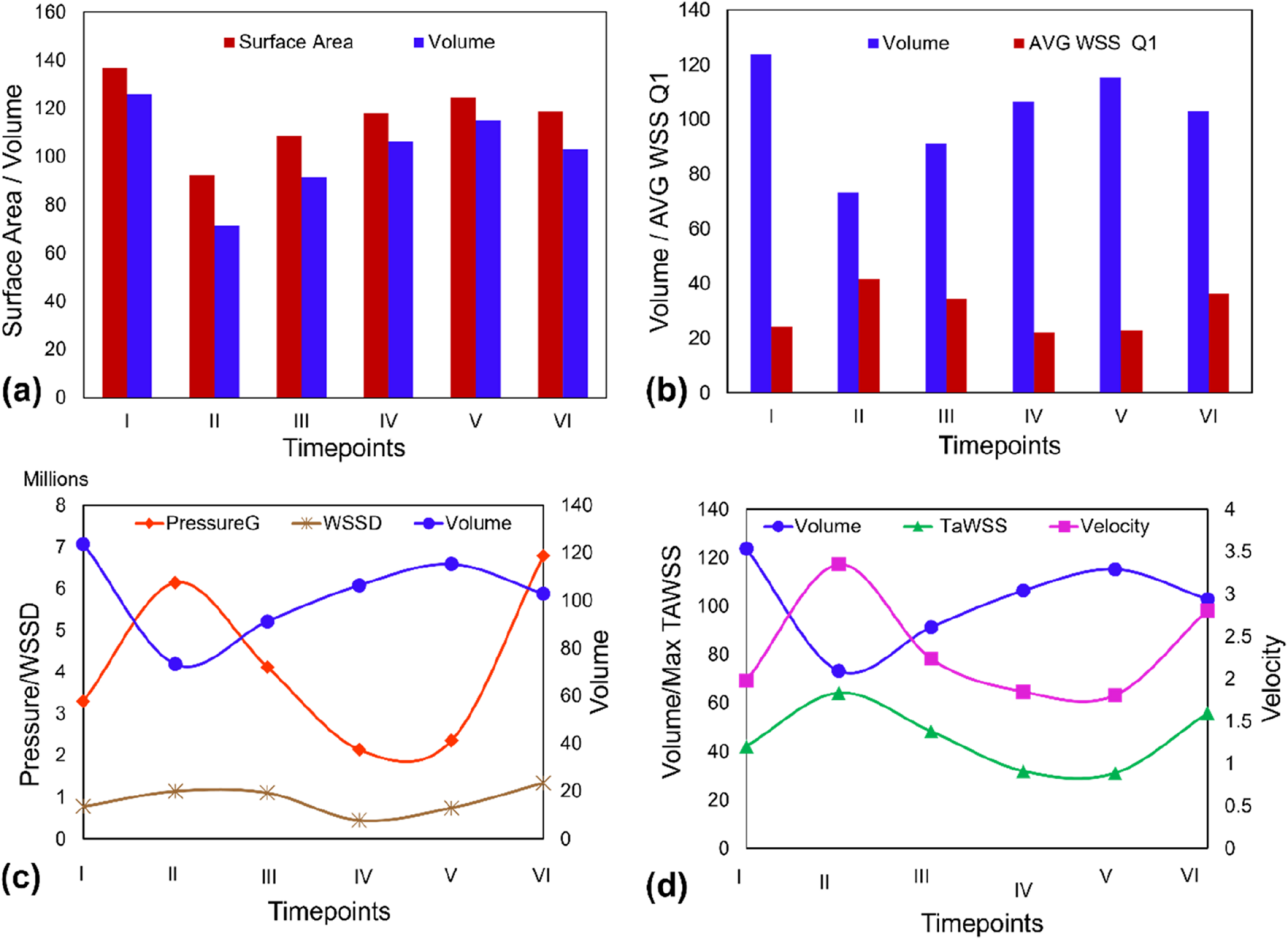
Morphology and hemodynamic comparisons of the aneurysm models reconstructed by operator D (**a**) Variation of surface area and volume for the aneurysm at different timepoints; (**b**) Correlation between volume and the average value of the first quartile of the WSS area; (**c**) Correlation between aneurysm volume, pressure gradient and wall shear stress divergence; (**d**) Correlation between aneurysm volume and maximum values of TaWSS and velocity.

The volume and surface area (SA) of the aneurysms has been measured at all six timepoints (Fig. 9a). It was observed that the volume and SA of the aneurysm decreased at timepoint II in comparison to timepoint I. From timepoint II–V the volume and SA of the aneurysm increased followed by a decrease from time point V–VI. It is important to note that a new daughter sac formed on the aneurysm at timepoint VI. This localised growth increased the risk of rupture and resulted in surgical intervention. Figure 9b shows that the volume and the average of WSS values in the first quartile are inversely correlated. Aneurysm volume is inversely correlated with pressure gradient as shown in Fig. 9c. This shows that the reduction in aneurysm volume increases the pressure. Similarly, the aneurysm volume is inversely correlated with the maximum values of velocity and TaWSS as shown in Fig. 9d.

## Discussion

Major factors involved in the formation, progression and rupture of aneurysm include the intracranial environment, mechanobiology, hemodynamic parameters and wall biomechanics^9,35–39^. Previous studies have shown that abnormal hemodynamic stresses promote size and shape changes in the aneurysm^40,41^. Therefore, with developments in computational resources, the use of numerical methods to investigate neurovascular diseases has increased in recent years. However, the results are not adopted in clinical practice due to the variation in results between different segmentation and simulation methods^29^. The use of the same protocol by different operators may influence the results attributed to an individual’s knowledge and experience. Therefore, this study evaluated the intra-team reproducibility of segmentation, model reconstruction and its influence on the CFD parameters. In addition, changes in morphological and hemodynamic parameters of the aneurysm over a decade were also evaluated to understand the interdependence of the size/shape changes and flow parameters.

According to the literature, the imaging technique used to reconstruct CFD models influences the hemodynamic parameters^23,24^. Segmentation methodology and post-processing steps also play an important role in assessing the clinically relevant morphological and hemodynamic parameters. Inter-team and intra-team variability also influence these processing steps, which affect simulation results^26,27^. In this study, although all operators used the same methodology and software, it was observed that there are differences in the geometries and lengths of the inlet and outflow branches. Minor variations in selecting the pixel intensity while segmenting and the smoothing applied to the geometries influenced the WSS and derived parameters. Also, the lengths of the inflow and outflow branches of the aneurysm influence the vessel wall area corresponding to the first quartile of the WSS values. The variability in geometry was observed around the aneurysm and the junction of the inlet and outflow branches. This variability is highly attributed to the level of smoothing and uneven surface smoothness resulting in sharp edges thereby increasing stress values. These geometrical differences resulted in a 20% variation in CFD results between operators. This agrees with the literature, with inter-team studies finding 28–51% variation in results^28^. The usage of a standardised protocol in this study minimised the variation in results and made it simpler to assess the variation between operators. Therefore, this study shows that standardised segmentation and reconstruction protocols can increase the reproducibility of the results. Additionally, as suggested in the literature, to ensure developed flow the operators should include the longest inflow and outflow vasculature possible^42,43^.

Previous literature and this current study show that the geometry of the aneurysm models influences the hemodynamic parameters^28,44,45^. Aneurysm geometry depends on imaging technique and quality, segmentation methodology, level of smoothing and manual post-processing of the surface mesh. In addition, it should be noted that intra-team variability influences these manual processing steps and vary the resulting model morphology and CFD parameters. Therefore, universal segmentation procedures could reduce the geometric variability thereby minimising the hemodynamic variability. Reducing the changes in morphological and hemodynamic parameters will globally increase clinicians’ confidence in the CFD simulations and can further be translated as a clinical tool to access the vulnerability of the aneurysm.

From the follow-up study on an aneurysm model of a single patient at six timepoints, there is a reduction in the size of the aneurysm from timepoint I to II which is more likely attributed to the blood-pressure-lowering treatment. However, from timepoint II to V there is a positive change in the aneurysm volume followed by a decrease at time point VI. At timepoint VI a new daughter sac has been formed thereby increasing the risk of rupture. This shape change aneurysm is associated with the co-existence of WSSD^+^ and WSSD^−^ in a circular pattern at timepoint V and more evidently at timepoint VI that might eventually result in rupture. Previous literature also suggests that the presence of the daughter sac significantly increases the risk of intracranial aneurysm rupture^33,46^. The aneurysm shape changes which occurred between timepoints V and VI were validated by the surgeons and led to surgical intervention for this patient. Aneurysm shape change with a smaller diameter is strongly associated with the risk of rupture and is treated as a high-risk aneurysm^47^.

The recirculatory flow in the aneurysm head and the associated changes in the WSS and WSS-derived parameters are due to changes in the morphological features. Therefore, both hemodynamic and morphological characteristics are important to identify the risk of aneurysm rupture^40^. This preliminary follow-up study showed that the direction and magnitude of WSS represented by WSSD appears to be a useful parameter to understand intra-aneurysmal flow mechanics and its influence on future morphological changes. Future large-scale follow-up studies are required to identify blended morphological–hemodynamic parameters for accurate risk assessment and patient-specific treatment procedures.

### Study limitations

This study has various limitations. The boundary conditions for the CFD analysis were assumed to be uniform across all the timepoints analysed, as only geometric information was available for the analysis. Hence, a waveform was assumed based on literature and mass flow rates in the carotid arteries. In future studies, the use of patient-specific boundary conditions would be more precise for analysing the hemodynamic parameters and their influence on the initiation, progression, and rupture of the aneurysm. Moreover, blood was assumed as a laminar, continuous Newtonian fluid as this study only focused on the reproducibility and influence of geometry changes on the CFD parameters and vice-versa. Finally, the aneurysm wall was assumed to be rigid. To improve simulated results, a dynamic mesh with a moving boundary approach to modelling the aneurysm wall should be considered.

The follow-up data of only one patient was analysed. Large-scale longitudinal studies are required to establish a correlation between geometry, and hemodynamic parameters and propose a WSS-derived parameter to estimate the vulnerability of the aneurysm. Additionally, this study design is limited to obtain general conclusions about intra-team reproducibility as the results of a limited number of operators (one experienced and 3 trained operators) were analysed. Although current reproducibility results show clear similarities and differences in certain parts of the process, these results will be further substantiated with the analysis of the results of more operators both experienced and trained.

## Conclusion

The reproducibility study emphasizes that intra-team variability highlights how operator-agnostic geometric variations impact CFD parameters. Small variations in the segmentation, level of smoothing and geometry reconstruction resulted in large differences in the WSS and derived parameters. Overall, there was an 80% reproducibility of the results, however, standardized segmentation and reconstruction protocols will increase the reproducibility of the results and clinician’s confidence in the CFD-based simulations. Follow-up imaging of the intracranial shape change aneurysm showed a strong association between the co-existence of WSSD^+^ and WSSD^−^ and the increase in the risk of rupture. Therefore, it is very important to keep track of the size and shape changes and corresponding CFD simulations will enable researchers to develop the correlation between morphological changes and subsequent hemodynamic changes and vice-versa. Moreover, there is scope to identify abnormal size and shape changes that help us identify high-risk aneurysms.

## Supplementary Information

Supplementary Figures.

## Data Availability

The data that support the findings of this study are available from the corresponding author upon reasonable request.
