# Supplementary material for: Reproducibility of the computational fluid dynamic analysis of a cerebral aneurysm monitored over a decade
Source: Sci Rep. 2023 Jan 5;13:219. doi: 10.1038/s41598-022-27354-w (PMC9816094; doi:10.1038/s41598-022-27354-w)
Supplement: Supplementary file 1 — Supplementary Figures. [file 41598_2022_27354_MOESM1_ESM.docx]

**Supplementary Material**

**Supplementary Figures:**

***
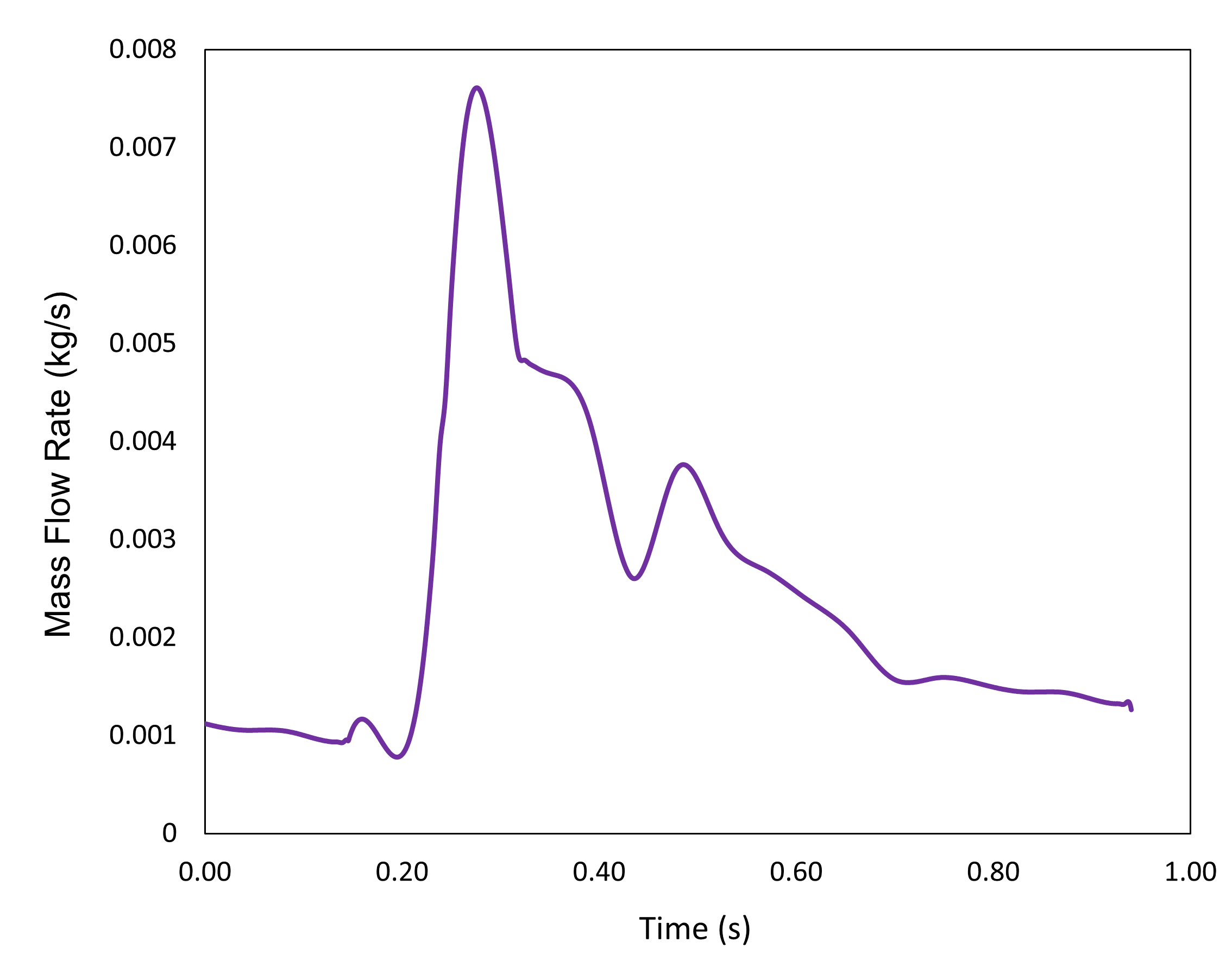
***

**Fig S1: Inlet mass flow rate profile used by all operators for all time points**


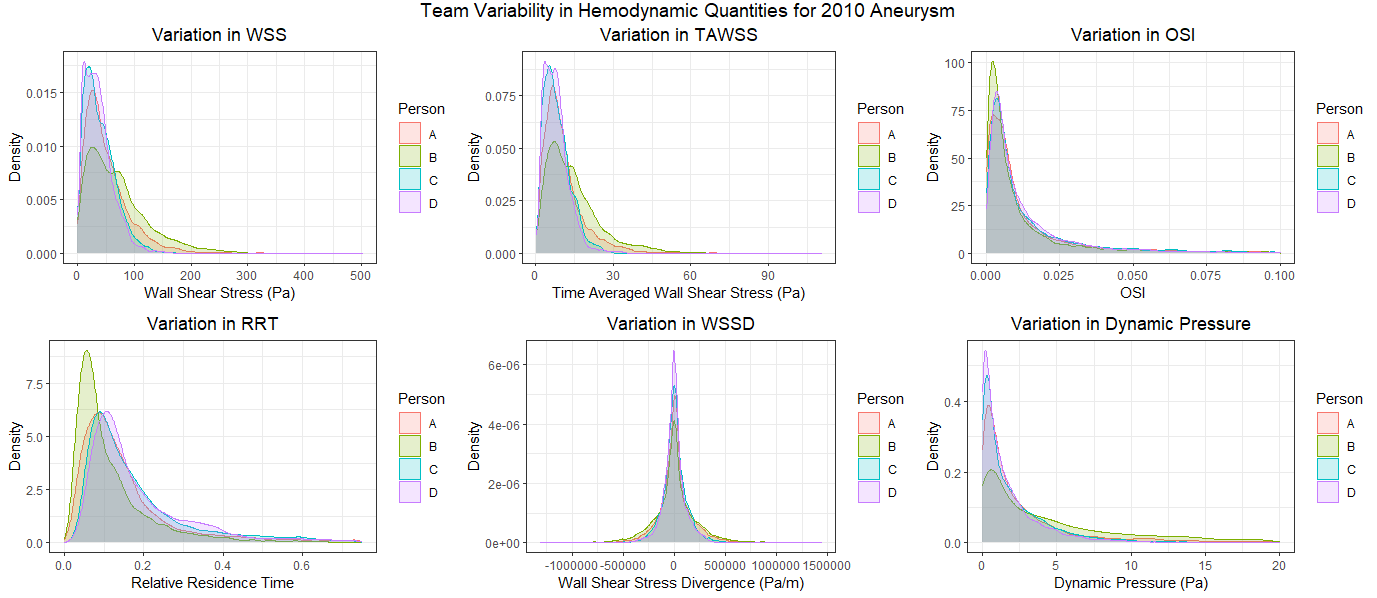


**Fig S2: Team variability in hemodynamic quantities for Timepoint I (2010)**. This figure illustrates the intra-team variability for different hemodynamic parameters for Timepoint I. The parameters include wall shear stress (WSS), time-averaged wall shear stress (TaWSS), oscillatory shear index (OSI), Relative Residence Time (RRT), wall shear stress divergence (WSSD), dynamic pressure (Pa).


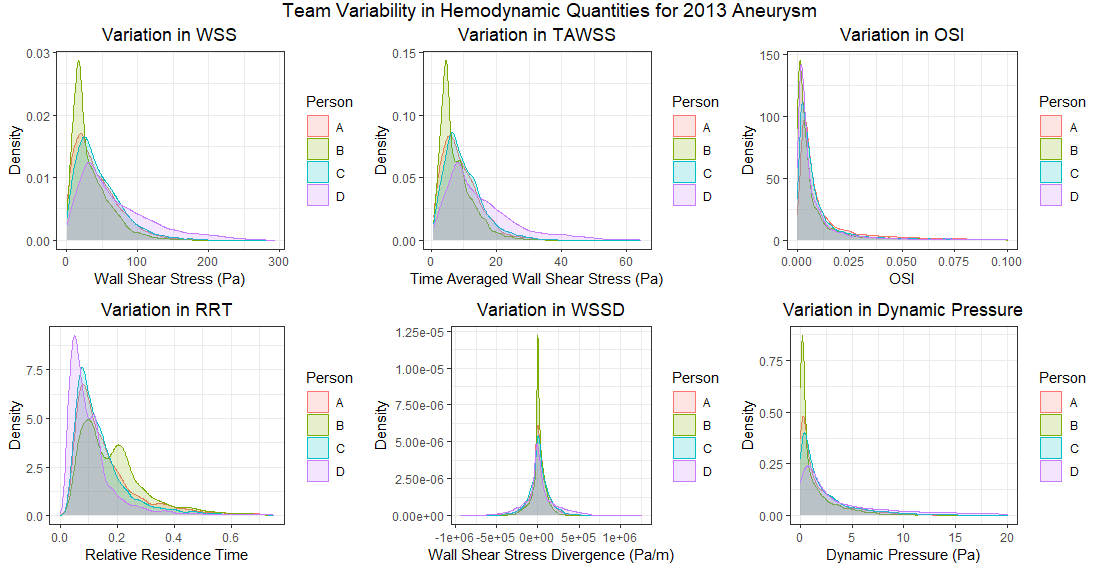


**Fig S3: Team variability in hemodynamic quantities for Timepoint II (2013)**. This figure illustrates the intra-team variability for different hemodynamic parameters for Timepoint II. The parameters include wall shear stress (WSS), time-averaged wall shear stress (TaWSS), oscillatory shear index (OSI), Relative Residence Time (RRT), wall shear stress divergence (WSSD), dynamic pressure (Pa).


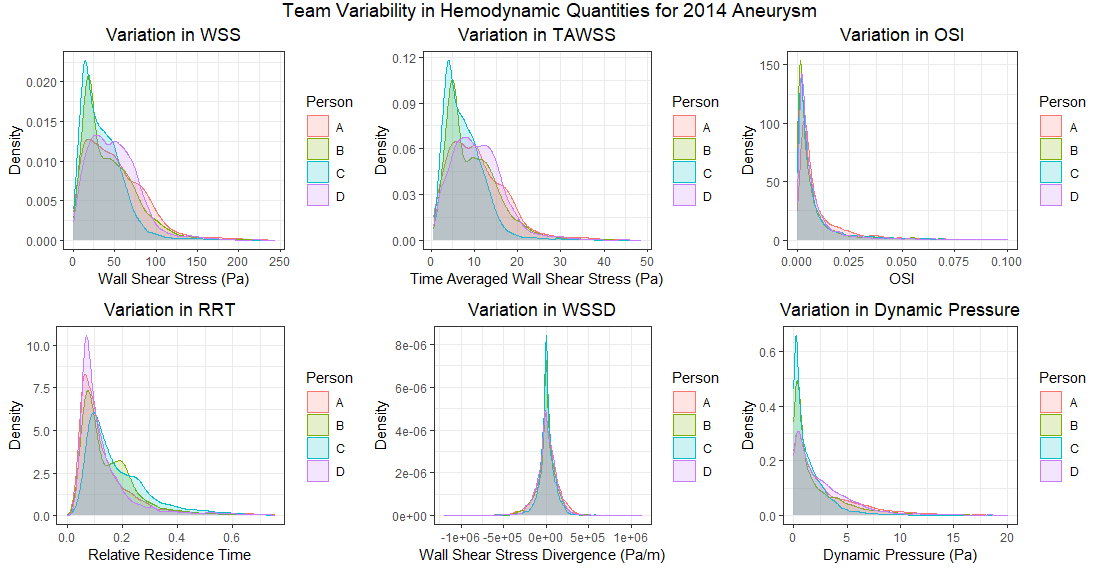


**Fig S4: Team variability in hemodynamic quantities for Timepoint III (2014)**. This figure illustrates the intra-team variability for different hemodynamic parameters for Timepoint III. The parameters include wall shear stress (WSS), time-averaged wall shear stress (TaWSS), oscillatory shear index (OSI), Relative Residence Time (RRT), wall shear stress divergence (WSSD), dynamic pressure (Pa).


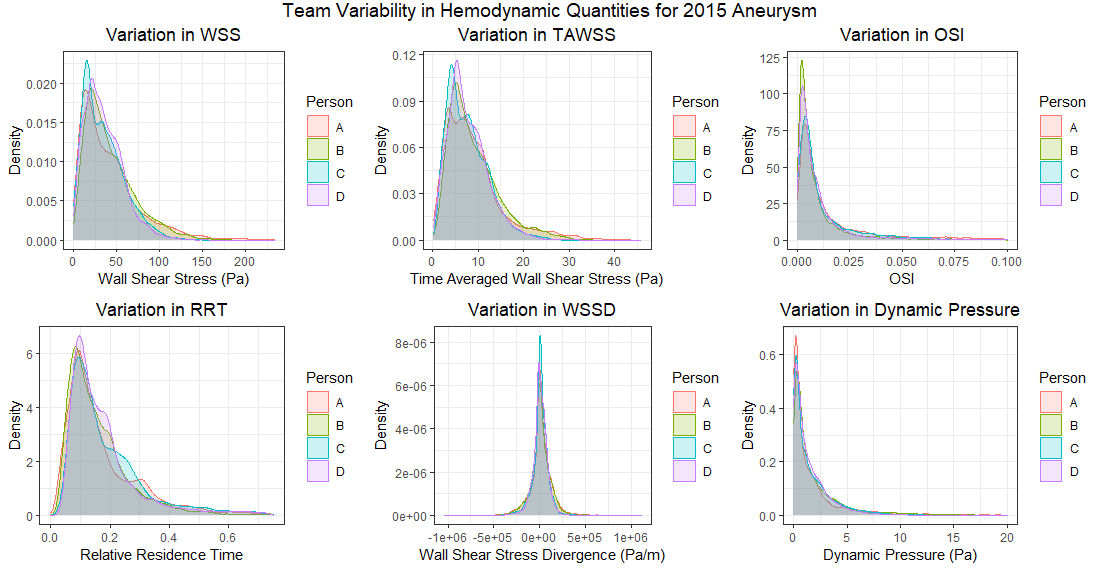


**Fig S5: Team variability in hemodynamic quantities for Timepoint IV (2015)**. This figure illustrates the intra-team variability for different hemodynamic parameters for Timepoint IV. The parameters include wall shear stress (WSS), time-averaged wall shear stress (TaWSS), oscillatory shear index (OSI), Relative Residence Time (RRT), wall shear stress divergence (WSSD), dynamic pressure (Pa).


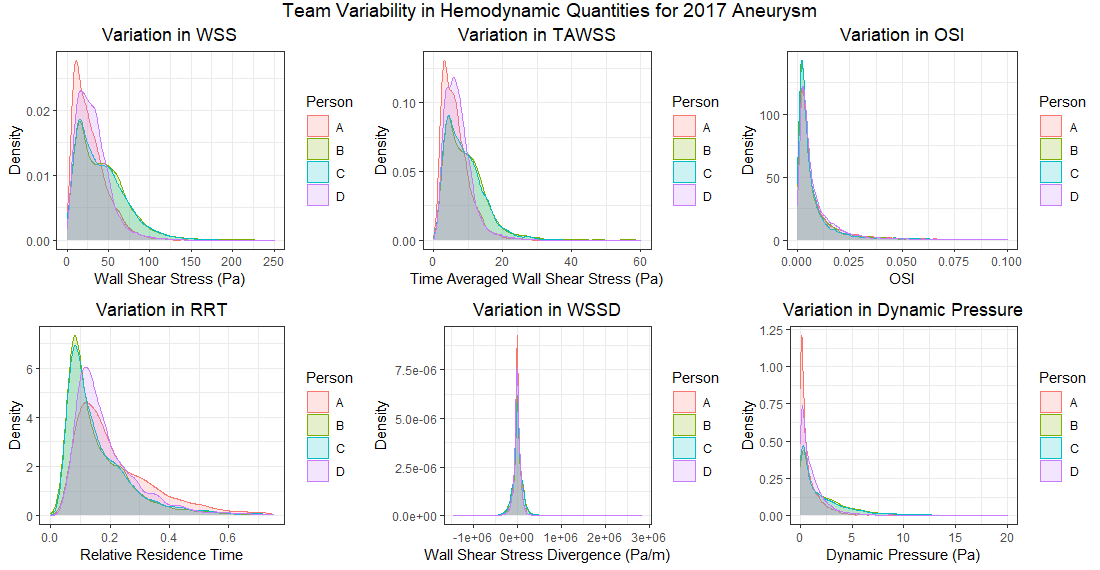


**Fig S6: Team variability in hemodynamic quantities for Timepoint V (2017)**. This figure illustrates the intra-team variability for different hemodynamic parameters for Timepoint V. The parameters include wall shear stress (WSS), time-averaged wall shear stress (TaWSS), oscillatory shear index (OSI), Relative Residence Time (RRT), wall shear stress divergence (WSSD), dynamic pressure (Pa).


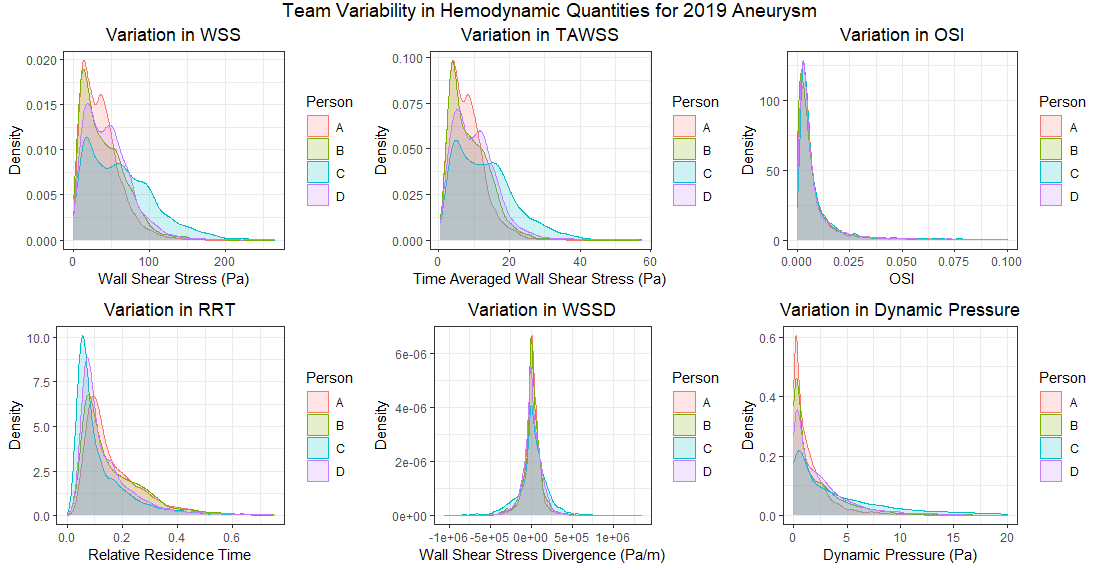


**Fig S7: Team variability in hemodynamic quantities for Timepoint VI (2019)**. This figure illustrates the intra-team variability for different hemodynamic parameters for Timepoint VI. The parameters include wall shear stress (WSS), time-averaged wall shear stress (TaWSS), oscillatory shear index (OSI), Relative Residence Time (RRT), wall shear stress divergence (WSSD), dynamic pressure (Pa).
